# Complete ectosome formation from the choanosome during whole-body regeneration of the sponge *Hymeniacidon heliophila* (Wilson 1911) using an in vitro microexplant model

**DOI:** 10.1007/s11626-026-01152-4

**Published:** 2026-02-20

**Authors:** Cristiano C. Coutinho, Fernando B. M. Henriques, Márcia Attias, Claudia Mermelstein, Manoel L. Costa

**Affiliations:** 1 Laboratório de Diferenciação Muscular, Instituto de Ciências Biomédicas, Universidade Federal Do Rio de Janeiro, Centro de Ciências da Saúde, room F2-019, Ilha Do Fundão, Rio de Janeiro, RJ Brazil; 2 Instituto de Biofísica Carlos Chagas Filho, Universidade Federal Do Rio de Janeiro, Rio de Janeiro, 21941-902 Brazil; 3 Instituto Nacional de Ciência e Tecnologia Em Biologia Estrutural e Bioimagens-INBEB, and Centro Nacional de Biologia Estrutural e Bioimagem-CENABIO, Universidade Federal Do Rio de Janeiro, Rio de Janeiro, RJ 21941-902 Brazil

**Keywords:** Epithelization, MET, Sponge, Regeneration, *Hymeniacidon heliophila*

## Abstract

The regeneration of the sponge *Hymeniacidon heliophila* was investigated using the in vitro microexplant model. This model differs from cell dissociation, surface ablation, and fragment culture models because it preserves the choanosome attached to the substrate, maintaining the apical-basal axis while entirely lacking the ectosome and the water flow polarity (ostia-osculum). The complete ectosome regenerative development was assessed using stereoscopy and scanning electron microscopy until complete adult formation. The observed stages of ectosome regeneration from the preserved choanosome align with existing data on sponge regeneration. Epithelization begins ubiquitously across the microexplant surface and expands to fully encase the microexplant. Two key processes were noticed: (1) the formation of an extracellular layer at the surface and (2) the appearance of discrete patches of pinacoderm over the extracellular matrix, probably by mesenchymal-to-epithelial transition. In more advanced regeneration stages, ectosomal spicules align transversely to the pinacoderm, with their tips protruding outward. These spicules provide structural support for the developing subdermal space while the choanosome concurrently becomes functional. Additionally, outer cells were identified in both adult microexplants and naturally growing individuals. Further efforts are needed to distinguish whether these outer cells are epibionts or autologous. As final considerations, the data were discussed from an evolutionary perspective that regeneration in sponges is an inherent feature linked to the emergence of multicellularity in animals. This process represents a primordial mechanism for stabilizing metazoan multicellular organization.

## Introduction

 Model organisms at the evolutionary base of animal origins, such as sponges, are appropriate for studying the transition from protozoa to animals. Comparative studies could reveal fundamental and common traits of programmed cellular processes involved in metazoan multicellular organization. For instance, the evolutionary origin of epithelization in animals is likely to be as ancient as the origin of animals themselves, representing a general property of living matter in multicellular homeostasis (Leys and Riesgo 2012; Elchaninov *et al*. 2021). Within this framework, epithelium formation during sponge regeneration is hypothesized to reflect an ancestral feature associated with the emergence of animal multicellularity. The present work aims to identify and characterize the basic and ancestral events of epithelium formation in animals. The identification of evolutionarily conserved traits would contribute to the development of bioengineered tissues and regenerative medicine.

Research on sponge regenerative processes has gained significant attention since dissociated cells were shown to spontaneously reorganize into a new individual (Wilson 1911; Korotkova 1972; Custódio *et al*. 1998). A compacted mass of dedifferentiated cells covered by a new epithelium-like structure, the primmorph, is assembled as a stage for somatic embryogenesis in demosponges (Ereskovsky *et al*. 2021). Under favorable conditions, primmorphs attach to the substrate, thus forming the basal–apical axis, and the aquifer system reestablishes the water flux axis (Müller *et al*. 2004; Chernogor *et al*. 2011; Ereskovsky *et al*. 2021). Dissociated sponge cells cultivated in FibraCel® disks, thin hydrogel layers, and gel microdroplets (GMDs) did not undergo morphogenesis and did not regenerate into adult sponges, but kept proliferating, in contrast to cells deprived of extracellular matrix adhesion (Urban-Gedamke *et al*. 2021). Dissociated sponge cells are appropriate for investigating sponge whole-body regeneration (WBR) but have the characteristic of complete disruption of all cell–cell and cell–matrix interactions.

Ablation at a specific site on the sponge surface (Wilson 1910; Alexander *et al*. 2015; Borisenko *et al*. 2015; Ereskovsky *et al*. 2020) evidenced (1) the closure of the injury and disintegration of structures in adjacent areas; (2) undifferentiated cell mass (blastema) formation; (3) epithelialization of the wound surface; and (4) the reorganization of the choanosomal structures according to the water flux axis (Borisenko *et al*. 2015).

Another model to investigate sponge regeneration, other than cell dissociation and superficial ablation, utilizes explants, which are fragments taken from adult sponges (Korotkova *et al*. 1983; Osinga *et al*. 1999; Hoffmann *et al*. 2003; Nickel & Brümmer 2003). In the fragment regeneration model, a similar sequence of programmed events develops, which are epithelialization of the wound surfaces and cell dedifferentiation with disintegration of the aquiferous system; and the fragment loses the original symmetry, becoming compacted, spherical, and radially symmetrical. As similarly reported in primmorphs in favorable condition, a fragment attaches on the substrate, the radial symmetry transit to apical‐basal symmetry, the aquifers system is restored, the water flux axis emerges, and the explant become less compacted with water permeability.

The microexplant approach reported here was first described by Custódio *et al*. (2002) and was an alternative method used in Coutinho *et al*. (2017) for in vitro investigation of cellular processes of the first-day regeneration in *Hymeniacidon heliophila*. The access of all techniques based on optics is the main contribution of the microexplant in vitro organotypic culture, thus being suitable for investigating sponges coordinated cellular processes. Microexplants are generated when a coverslip pair is taken from the inside of a natural living sponge, and the pair is opened to separate the two coverslips. Each pair is expected to contain sponge tissue attached to its external face, since an encrusting sponge grows over any natural substrate such as the silicate in a glass coverslip. The silicate from natural rocks and from coverslips is equivalent, and no differences in sponge morphology caused by these equivalent substrates are expected. However, we cannot exclude the possibility that differences in coverslip and granite rock substrates could influence microexplant morphology. Further experiments are needed to study this process. Some parts of the microexplant are composed of a monolayer of epithelial basopinacoderm attached to the coverslip by an extracellular matrix. Other areas of the microexplant display distinct thickness of preserved choanosome overlying the basopinacoderm. The microexplant is thus defined as a millimeter-sized sample of the preserved internal region (choanosome) attached to a coverslip, which has the capacity to form a new individual in vitro. Since the microexplant is generated inside the sponge body, it is devoid of the ectosome (a superficial tissue-like structure). This approach introduces intermediate stress by preserving the internal organization. The basal layer attached to the substrate is preserved, and it orients the apical–basal axis. The microexplant model does not cause the extreme structural disruption of complete cell dissociation, since apical–basal axis and extracellular–cell and cell–cell interactions are preserved. In contrast, the microexplant model does not cause the minor disruption generated by topical surface ablation, since topical surface ablation preserves (1) the apico–basal axis, (2) the water flux axis, and (3) most epithelial surface structures. Therefore, the microexplant is now used for in vitro investigation of the epithelization process without the contribution of any preserved ectosome or the water flux axis.

The epithelial and mesenchymal cell types of sponges may have originated through the diversification of flagellated and amoeboid cell states described in colonial choanoflagellate-like ancestors respectively (Brunet *et al*. 2021). Epithelial/mesenchymal classification is a primordial, ancestral feature of all animals and was adopted in this work to further discuss evolutionary aspects of the regenerative process. Moreover, specific cell types in sponges are hard to identify by transmission electron microscopy (TEM), fluorescence microscopy, or in situ hybridization. Only very remarkable cell types could be distinguished in our previous work using microexplant. For instance, the distinguished epithelial cells are choanocytes with choano and flagellum and the flattened pinacocytes in different locales (basal, internal, or superficial). The identified mesenchymal cells are amoeboid cells and spherical cells. 

The sponge epithelial cells are responsible for classical metazoan epithelial functions, but some divergent traits in sponges have been noticed. For instance, choanocytes form choanocyte chambers to promote changes in the environmental water. They also have stemness propriety (Funayama 2018), but their derived differentiated cells are not exclusively epithelial cells as in metazoans. Basopinacocytes promote substrate adhesion and apical-basal body axis, a function not common in metazoan epithelium. Endopinacocytes line the internal aquiferous channels of the choanosome, and the exopinacocytes form the exopinacoderm, the most external layer covering the sponge body (Simpson 1984; Boury-Esnault and Rützler 1997; Ereskovsky and Lavrov 2021). The sponge mesenchymal cells are located within the mesohyl, wrapped in a collagen extracellular matrix. Archaeocytes, known for their intensive phagocytic activity, show stemness characteristics for generating epithelial and mesenchymal cell types (Funayama 2018). Demosponges do not have a specific stemness system for epithelial and mesenchymal cells but rely on the plasticity of transdifferentiation among mesenchymal and epithelial cell types. Other mesenchymal cells are terminally differentiated, such as sclerocytes, for siliceous spicules formation; collencytes and lophocytes, which produce the collagenous extracellular matrix (spongin); and spherulous cells, some of which store chemical defenses, and cells with various other inclusions (Simpson 1984; Boury-Esnault and Rützler 1997; Ereskovsky and Lavrov 2021). The lack of specific stem cell system for demosponges’ epithelial and mesenchymal cells, together with the huge plasticity of cell type transdifferentiation, are specific demosponge traits modulating sponge regeneration. Extracellular matrix deposition and fast formation of an epithelial covering layer over the surface are common traits in metazoans. Both evolutionarily conserved and derived traits were here considered to address the evolutionary origin of surface regeneration in metazoans. 

The novelty and primary aim of this study are to investigate the epithelization process without the contribution of any preserved ectosome and water flux axis. We expect to confront the data from microexplant epithelial regeneration with primmorph, superficial ablation, and explant regeneration data. This approach could identify basic and ancestral events of the formation of the epithelium in animals.

## Material and methods

### Sponge selection

*Hymeniacidon heliophila* has a 1–3 cm-thick base and uneven digitations that rise from a few millimeters to 4 cm high. Several attributes make *Hymeniacidon heliophila* a model organism to investigate regenerative development in sponges. For instance, (1) this specific sponge has characteristics for high regenerative performance, as it belongs to the demosponge class; (2) it is an encrusting sponge with bidimensional growth that is easier to measure; (3) it is highly tolerant to epibionts, debris, and microorganisms in tropical eutrophic bay water from the metropolis of Rio de Janeiro, thus simplifying the cultivation process; (4) it is resistant to intertidal variation stress (Wulff 2006; Coutinho *et al*. 2017; Ascer *et al*. 2024), contributing to easier booth cultivation and manipulations; (5) it is appropriate to generate in vitro regenerating microexplants (Coutinho *et al*. 2017).

### Microexplant generation

The microexplants were generated following the method described by Coutinho *et al*. (2017). Briefly, two coverslips were placed in contact with each other and inserted into the choanosome of a wild-type *Hymeniacidon heliophila* sponge that was naturally attached to rocks in Guanabara Bay, Rio de Janeiro, Brazil. After 3 days inside the sponge, the coverslips were retrieved. This duration was sufficient for the choanosome to attach to one face of each coverslip. Since an encrusting sponge grows over any natural substrate, such as glass coverslips and silicate rocks, no influence from the coverslip glass material is expected. Microexplant formation began, and regenerative development commenced when the coverslips were removed from the sponge body. Some areas of the coverslips retained the choanoderm of the native sponge, while others had only the basoepithelial layer attached, and some areas were devoid of sponge tissue entirely.

All microexplants were regenerated on a platform submerged 2 cm into an aquarium, with a continuous flow of surface water and uninterrupted lighting. Daily, a plastic pipette was used to flush aquarium water over the microexplants to remove debris. The aquarium conditions were adapted from Wanick *et al*. (2013, 2015), who reported a syntrophic environment favorable for cultivating sponges from eutrophic waters in Rio de Janeiro. The temperature of the aquarium water was maintained between 23–28°C.

### Follow up of regenerating microexplants

The regenerative development of 26 microexplants was photographically recorded using a Leica M205 FA (Wetzlar, Germany) stereoscopic microscope  on days 0, 1, 2, 4, 8, 12, 15, and 22 of regeneration. Before imaging, debris and algae were removed using forceps under the stereoscopic microscope. Successful regeneration was defined by the emergence of adult morphological traits, starting with the development of a more water-permeable choanosome, followed by the formation of a main water channel connected to a functional osculum.

### Scanning electron microscopy

Microexplants were generated as described above, and at least one microexplant was fixed on days 0, 1, 2, 3, 7, and 24 of regeneration. Fixation was performed using 2.5% glutaraldehyde in 0.1 M sodium cacodylate buffer (pH 7.3). The samples were then rinsed three times for 10 min each in the same buffer, followed by post-fixation in 1% OsO₄ plus 0.8% potassium ferrocyanide in 0.1 M cacodylate buffer for 30 min at room temperature in the dark. After post-fixation, the samples were rinsed twice in the same buffer and then in distilled water. Dehydration was carried out using progressively increasing concentrations of ethanol, as described previously (Magno *et al*. 2005).

Following dehydration, the coverslips with the samples were critical point-dried in CO₂ using a Leica 300CPD (Leica, Wetzlar, Germany), mounted on aluminum stubs, and sputter-coated with a 10 nm platinum layer using a Q150V Plus Quorum Sputter Coater (Sputter Coater, Lewes, UK). The samples were observed either in a Quanta 250 SEM (Thermo Fisher Scientific, Waltham, Massachusetts) with a tungsten filament (Thermo Fisher) or in a Quattro SEM (Thermo Fisher Scientific, Waltham, Massachusetts) with a field emission gun, operating at 5.0 kV and a 10-mm working distance. The background of the photos, symbols, and color adjustments to highlight specific structures were processed using Adobe Photoshop (Adobe Systems Inc., San Jose, CA, Waltham, MA).

## Results

### Microexplant regeneration to the adult stage

The formation of a new adult from the choanosome begins when the coverslip is removed from the inner region (choanosome) of *Hymeniacidon heliophila*. The first 8–24 h were already described by Coutinho *et al*. (2017), which demonstrated the choanosomal structure spreading several millimeters (centrifugal displacements) over the adjacent regions with only a basoepithelial layer. The subsequent event within the first 24 h involves the dissembling of the choanoderm and collective migration of most cells, oriented toward one or two primary cell clusters (centripetal migration) (Coutinho *et al*. 2017). The clusters have dense cell organization representing resistant states similar to asexual reproduction gemmules and stress resistant forms like primmorfs. Dense microexplants exhibit a homogeneous orange/yellow coloration, lack a subdermal space (or have only a minimal space), and contain a central mass of mesenchymal cells covered by pinacoderm.

**Figure 1.**
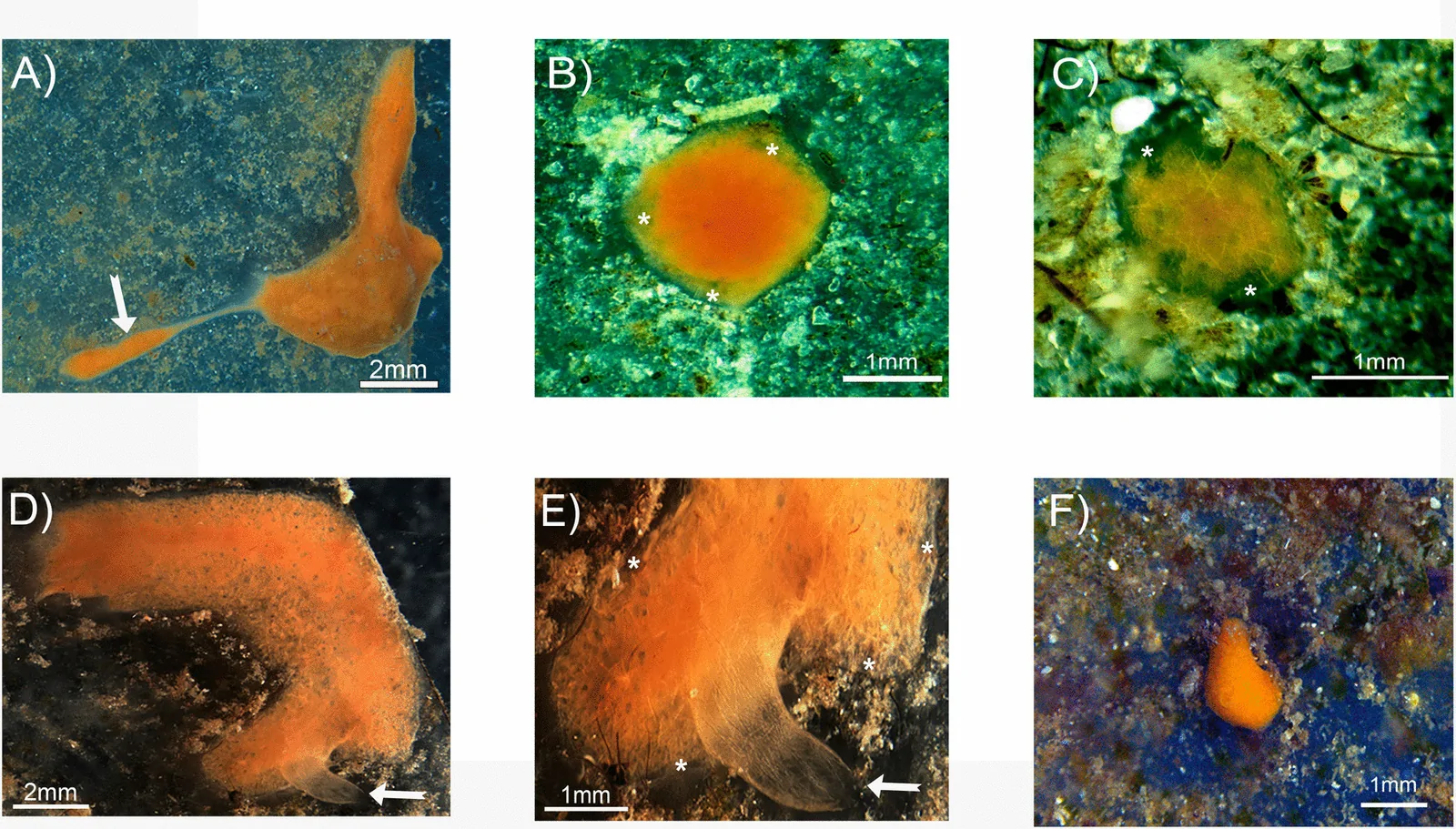
Regenerative stages of the microexplant. (*A*) A 3-d-old microexplant showing streams of collectively oriented cell migration toward the nearest cell cluster. The formation of a secondary cell cluster is indicated by an *arrow*. (*B*) An intermediate state of a 3-d-old microexplant, showing a still condensed cell mass, but with subdermal space (*). (*C*) The smallest microexplant in the adult stage (7 d), containing a water channel-rich permeable structure and subdermal space (*) but lacking an osculum. (*D*) An adult microexplant after 8 d of regeneration, displaying a water channel-rich choanosome forming a permeable structure. The osculum is indicated by an *arrow*. (*E*) A magnified view of the osculum shown in *D*, indicated by an *arrow*. The subdermal space is indicated by (*). (*F*) A condensed cell mass after 3 d of microexplant regeneration, resembling reduction bodies. Under stressed conditions, it is devoid of feeding features such as water channels, subdermal space, and an osculum.

To further extend the investigation in this work beyond the first 24 hours, 26 regenerating microexplants were recorded periodically with a stereomicroscope (Fig. 1). According to the data, the cell mass of the microexplant can remain in this contracted, reduced-body stage for several d (Fig. 1*A*, *F*), becoming more spherical as substrate adhesion decreases over time, or can progress to the adult stage (Fig. 1*A*–*E*). Typically, 8-d microexplants at the adult stage are characterized by the emergence of water channels and other feeding structures, such as a permeable choanosome (Fig. 1*C*–*E*), subdermal space (Fig. 1*B*–*E*), and an osculum (Fig. 1*D*, *E*). These feeding structures indicate a functional state. The inflated young osculum, made of thin walls and dominated by aligned spicules in poor organic tissue (Fig. 1*E*), is an indication of a functional osculum. Water pumping activity through the osculum was indeed observed (data not shown). Under unfavorable conditions, the microexplant contracts further, becomes less water permeable, loses the feeding structures of the adult stage, and is remodeled by centripetal cell movements (Fig. 1*F*). A second transition, with a return to adulthood, has not been tested, but is expected. The adult stage is consistently observed only under favorable aquarium conditions. The ostia for water inflow are only observed by scanning electron microscopy (SEM) and will be shown further on. No significant volume increase was noted during and after reaching the adult stage.

### Microanatomy of the regenerating ectosome

**Figure 2.**
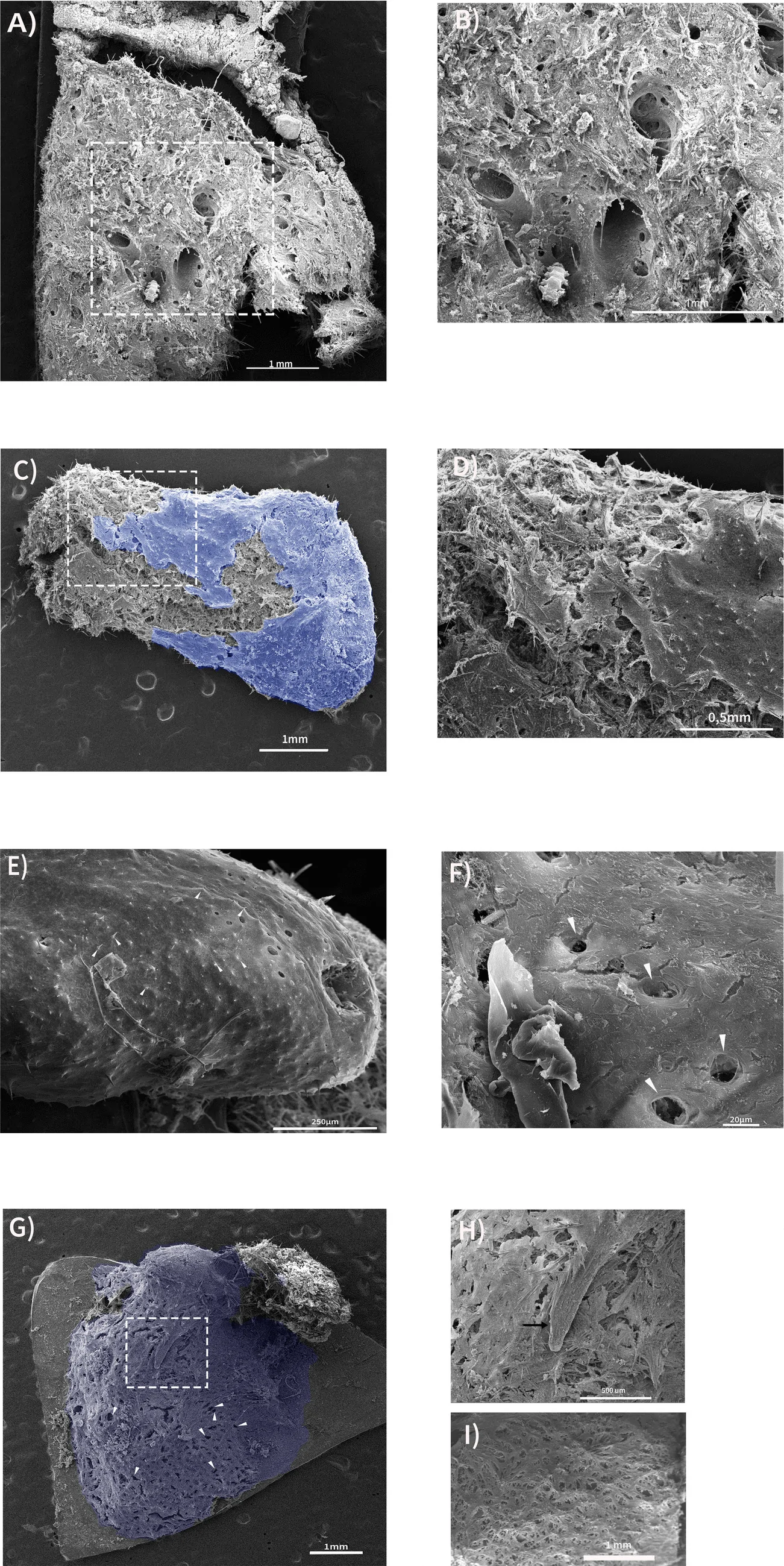
Scanning electron microscopy (SEM) of pinacoderm formation. (*A*) A 0-h microexplant lacking exopinacoderm at the initial stage of the epithelialization process. (*B*) Inset from *A* evidencing the absence of flattened exopinacoderm, but projecting spicules and exposed extracellular matrix with some endopinacocyte forming water channels. (*C*) A 1-d-old microexplant with large regions digitally highlighted in light blue to emphasize the patches of exopinacoderm epithelial layer. The uncolored regions remain devoid of pinacoderm. (*D*) Inset from *C* showing the border line between exopinacoderm-covered and uncovered regions. (*E*) Seven-d-old microexplant with formed pinacoderm showing regular pattern of spicules pointing to the exterior (*arrowhead*). (*F*) Close-up view of ostia formed in the pinacoderm of a 7-d-old microexplant (indicated by *arrowheads*). Breaks in continuous pinacoderm are probably artificially caused by the condition of SEM fixation. (*G*) Some 3-d-old microexplants were not fully covered by pinacocytes (digitally highlighted in *light blue*), and some exopinacoderm gaps were noticed, as indicated by *arrowheads*. (*H*) Inset from (*G*) pointing by a black arrow to a putative region with collective cell migration and oriented spicules. (*I*) Similar stages of exopinacoderm with gaps, which are devoid of basing fibrous extracellular matrices, were also noticed in one-d-old microexplants.

The analysis by stereomicroscopy evidenced a very early formation of an epithelial cover over the microexplant after 1–3 d of regeneration. To further extend the investigation of ectosome formation from the choanosome, scanning electron microscopy (SEM) was used to analyze the morphological progression of the ectosome structures towards the adult stage. As shown in Fig. 2, epithelialization of the explants began on day 0, but no exopinacoderm is present at this time (Fig. 2*A*, *B*). Patches of pinacoderm formation were identifiable by day 1 (Fig. 2*C*–*D*), indicating the lack of an origin center, but a mosaic-like formation of the pinacoderm. Over the course of epithelial regeneration, exopinacoderm covered the microexplant surface with the formation of ostia. A 7-d-old microexplant completed the ectosomal regenerative process, as indicated by the continuous exopinacoderm with ostia and regularly organized spicules projecting to the exterior (Fig. 2*E*, *F*). However, some microexplants on day 3 were not fully covered by pinacoderm and some gaps remained (Fig. 2*G*–*I*). These gaps in the pinacoderm lack flattened pinacocytes and are devoid of underlying extracellular matrix.

**Figure 3.**
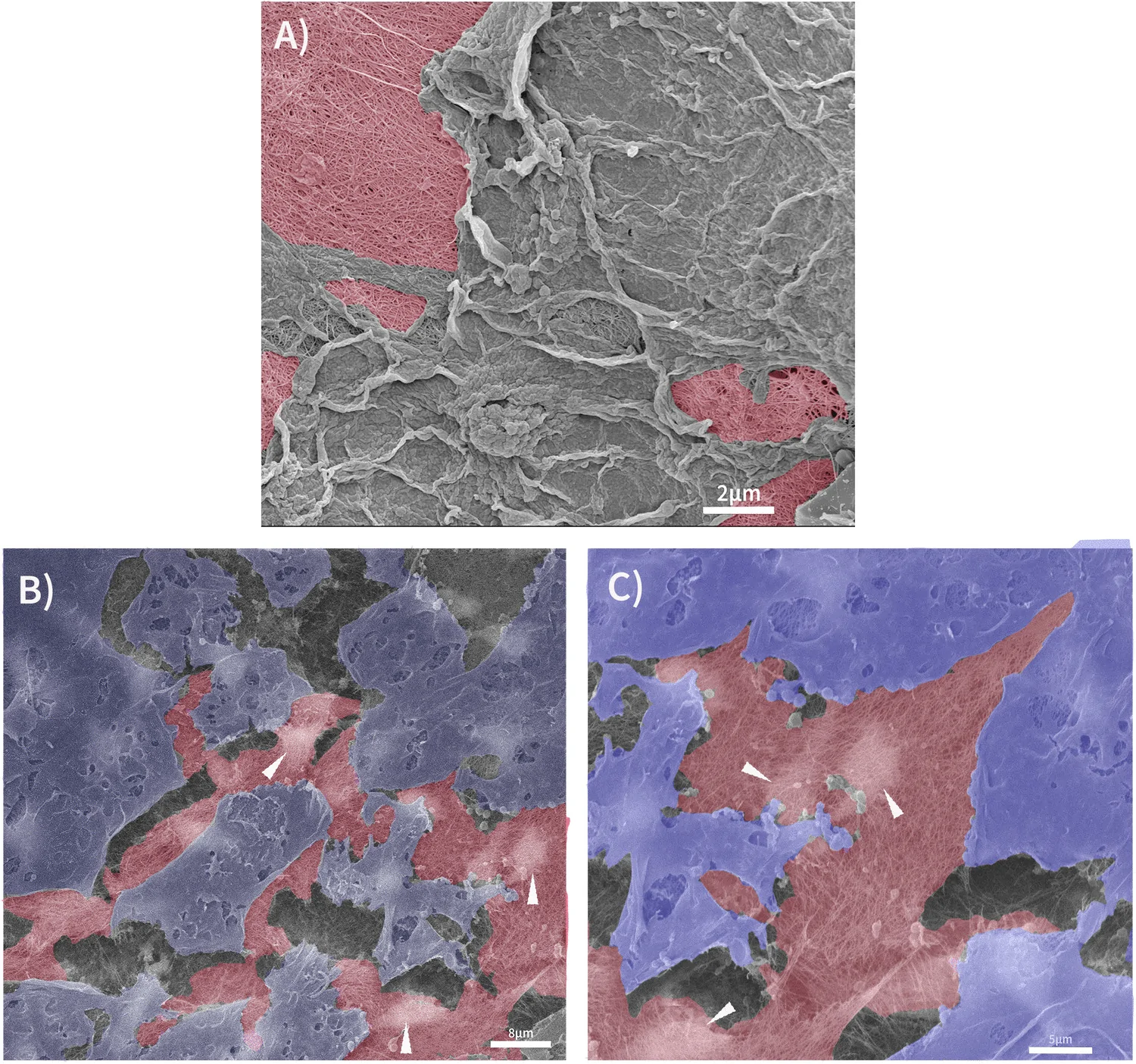
Scanning electron microscopy (SEM) of pinacocyte layer over the extracellular matrix for pinacoderm formation. (*A*) The external surface of a 1-d-old microexplant, with a digitally colored region in red highlighting the details of collagen fibers forming a two-dimensional matrix. The grey region depicts pinacocytes migrating over the matrix. (*B*, *C*) One-d-old pinacoderm, composed of collagen fibers (digitally highlighted in *red*) underlying the pinacocyte layer (digitally highlighted in *blue*). The gaps between the blue-colored exopinacocytes exposed immersed nucleus (indicated by *arrowheads*) embedded within the underlying collagen mesh, probably indicating “T” shape exopinacocytes. Disruptions of cell membranes are artificially caused by the condition of SEM fixation.

The collagen extracellular matrix is initially deposited within the mesenchymal cell mass, after which pinacocytes migrate across this superficial collagen matrix (highlighted in red in Fig. 3) or emerge from the mesohyl. The flattened superficial pinacocytes over the partially exposed fibrous extracellular matrix indicate an intermediate stage of pinacoderm regenerative development. The final pinacocyte “T” morphology, in which the nucleus is immersed in the extracellular matrix and the flattened cell body covers the surface (Simpson 1984; Boury-Esnault and Rützler 1997), is expected at the final stage. The presence of a cell nucleus immersed in the fibrous mesh of the extracellular matrix may indicate T-shape morphology, but the data are not conclusive (Fig. 3*B*, *C*).

### Transversely oriented spicules projecting over the pinacoderm layer

**Figure 4.**
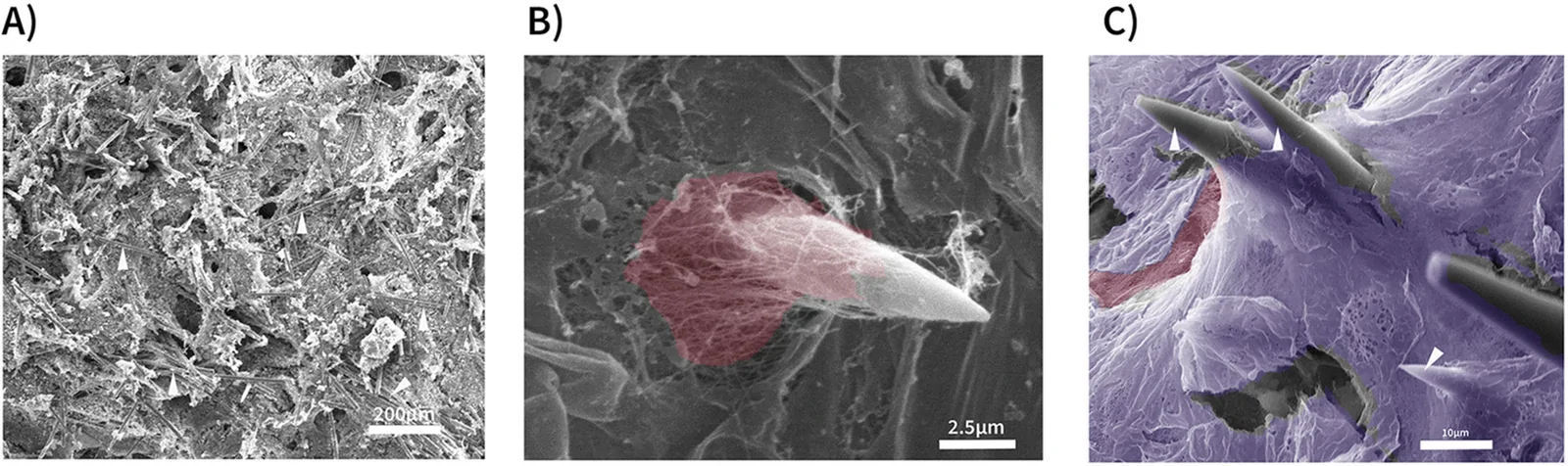
Scanning electron microscopy (SEM) showing pinacoderm formation (epithelialization) around spicules. (*A*) Surface of a 0-h microexplant displaying disorganized spicules (indicated by *arrowheads*) in regions lacking pinacoderm. (*B*) Close-up of a spicule anchored by collagen fibers (digitally highlighted in *red*) within the pinacoderm of a 7-d-old microexplant. (*C*) Advanced epithelialization stage with pinacocytes (digitally highlighted in *blue*) covering and anchoring transverse spicules (indicated by *arrowheads*) in a 3-d-old microexplant. The underlying collagen mesh is digitally highlighted in red. Breaks in continuous pinacoderm are artificially caused by the condition of SEM fixation.

The mineral skeletal pattern of the ectosome was at least partially restored when spicules were arranged transversely through the exopinacoderm layer, with their tips projecting regularly across the pinacoderm cell layer. The majority of spicules were rearranged and not all ectosomal spicules were turned due to the short duration of the experiment. Collagen was initially observed at the tips of these spicules, after which pinacocytes covered this fibrous collagen layer (Fig. 4). This pattern was observed in all samples from day 1 to day 7, with different developmental stages present within the same sample. By day 24, all samples had fully completed epithelialization.

### Subdermal space formation

**Figure 5.**
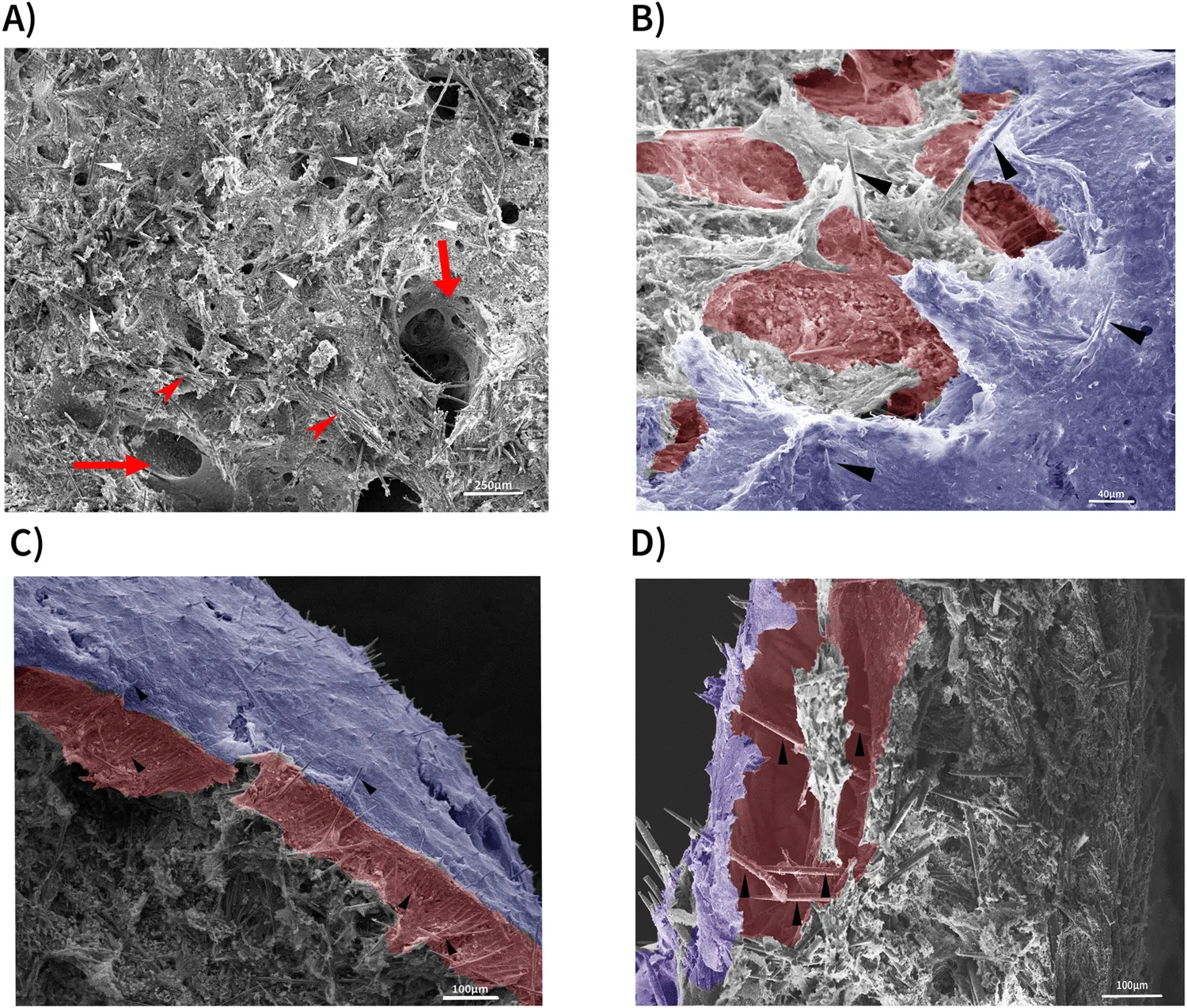
Scanning electron microscopy (SEM) of the ectosome, illustrating the formation of subdermal space during the regenerative process. (*A*) A 0-h microexplant with an exposed choanosome composed of thick-walled mesohyl and water channels. The mesohyl contains disorganized spicules (white *arrowhead*) embedded in the extracellular matrix, along with adherent mesenchymal cells. Two spicule bundles are visible (red *arrowhead*). Water channels lined by epithelial endopinacocytes are indicated by red *arrows*. (*B*) A 1-d-old microexplant undergoing epithelialization, with pinacocytes (digitally highlighted in *light blue*) spreading over the mesohyl (digitally highlighted in *red*). Spicules are disorganized, with some spicules projecting transversely toward the exterior (*arrowhead*). (*C*) A 2-d-old microexplant with established subdermal water channels (digitally highlighted in *red*) covered by pinacoderm (digitally highlighted in *blue*). Surface spicules are organized and projecting outward in a regular pattern (*arrowhead*). Breaks in the continuous pinacoderm are caused artificially by the condition of SEM fixation. (*D*) A 20-d-old microexplant with fully formed subdermal water channels (digitally highlighted in *red*), pinacoderm (digitally highlighted in *light blue*), and spicules projecting outward in a regular pattern (*arrowhead*).

**Figure 6.**
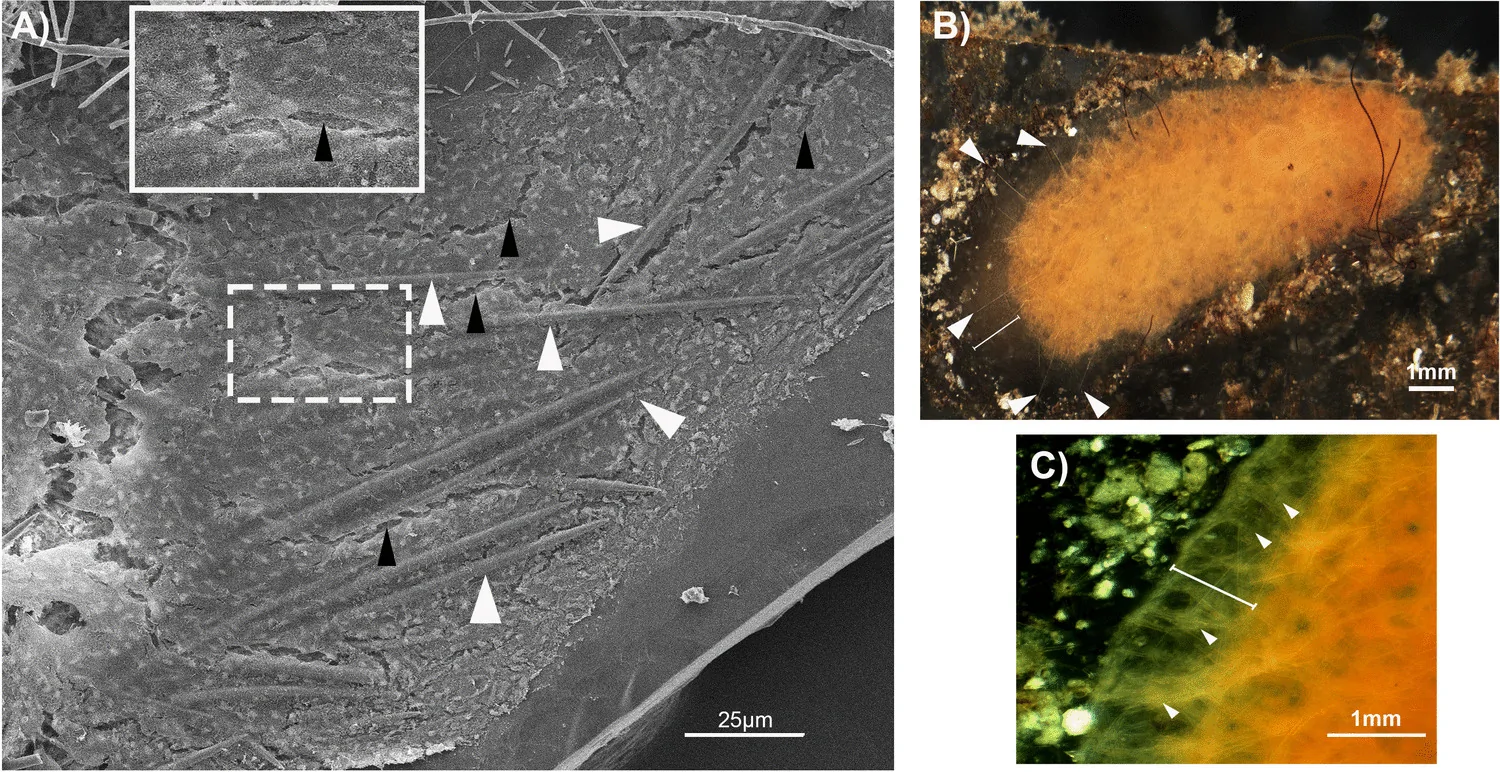
Developmental progression of the subdermal space. (*A*) Edge surface of a 3-d microexplant with a spicule-thick mesohyl layer. The absence of a fully developed ectosome is evident at this peripheral region of the sponge microexplant. Subdermal spaces and choanoderm are not yet present. Cracks along the flat pinacoderm reveal a scarce or poorly apparent fiber-bearing base layer of the extracellular matrix (*black arrowhead*), which maintains the spicules oriented horizontally (*white arrowhead*). Dashed box was magnified in the *white box* to show the scarce and poorly apparent reticular organization of the extracellular matrix pointed by the *black arrowhead*. Breaks in the continuous pinacoderm are artificially caused by the condition of SEM fixation. (*B*) 12-d adult microexplant with transversely oriented spicules (*arrowhead*) supporting the subdermal space, as indicated by the white line. (*C*) Magnified view of a more developed subdermal space, showing transversely oriented spicule meshes (*arrowhead*) supporting the subdermal space, as indicated by the *white line*.

The final stage of epithelialization involved the formation of a subdermal space (Fig. 5). By day 2, a subdermal water channel was visible in certain areas beneath the pinacoderm. At this stage, the peripheral spicules were already organized transversely to the sponge surface, with their tips projecting outward in a regular pattern. The transverse arrangement of these peripheral spicules is likely essential for the formation of the subdermal water channel, as discussed later. In regions lacking a subdermal space, the spicules remain horizontal and are expected to become transverse following colonization by mesenchymal cells (Fig. 6).

### Outer cells

**Figure 7.**
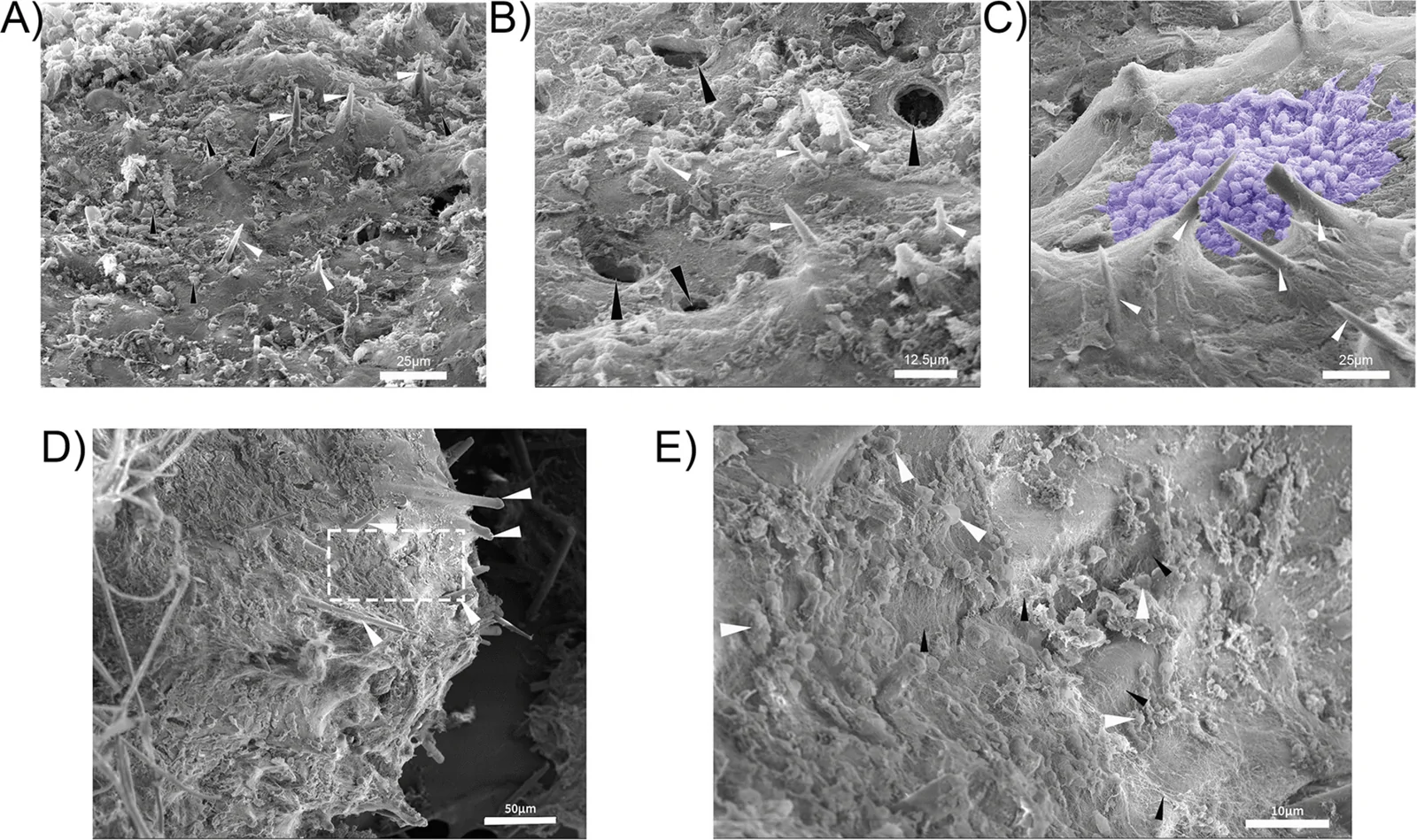
Scanning electron microscopy (SEM) of the external surface of the pinacoderm in *Hymeniacidon heliophila* grown under natural conditions and in a 3-d-old regenerating microexplant. (*A*) Low-magnification view of the pinacoderm from a *Hymeniacidon heliophila* sample collected from the rocky coast, showing transversely oriented spicules (*white arrowhead*) projecting outward and cells (*black arrowhead*). (*B*) Mesenchymal sponge cells, or epibionts, spread across the surface of the pinacoderm in the rocky coast sample. Ostia (*black arrowhead*) and projecting transversely oriented spicules (*white arrowhead*) are visible. (*C*) Sponge mesenchymal cells, or epibionts (digitally highlighted in *light blue*), clustering on the pinacoderm of a 3-d-old regenerating microexplant. Transversely projecting spicules are indicated by *white arrowheads*. (*D*) Sponge mesenchymal cells, or epibionts, on the external surface of the pinacoderm in a 24-d-old regenerating microexplant. Transversely projecting spicules are visible in the image (*white arrowheads*). (*E*) Magnified view of the dashed square in image D, highlighting spread sponge mesenchymal cells, or epibionts (*white arrowhead*), on the pinacoderm. A mesh of collagen fibers (*black arrowhead*) is visible over the exopinacoderm.

Mesenchymal crawling cells, resembling autocrine mesenchymal cell types, were observed outside the wild-type non-cultivated control samples. Alternatively, epibionts are also possible, and the images cannot distinguish them. Further investigations are necessary to resolve the issue. These cells were found outside the main body, adhering to the external surface of the exopinacoderm, as shown in Fig. 7*A*–*B*, for naturally growing control individuals. A similar phenomenon was observed in regions of microexplants with fully sealed exopinacoderm (Fig. 7*C*–*E*). Two distinct cell organizations were identified on the exopinacoderm: cell clusters and spreading amoeboid cells.

### Summarized stages of microexplant ectosomal regeneration

**Figure 8.**
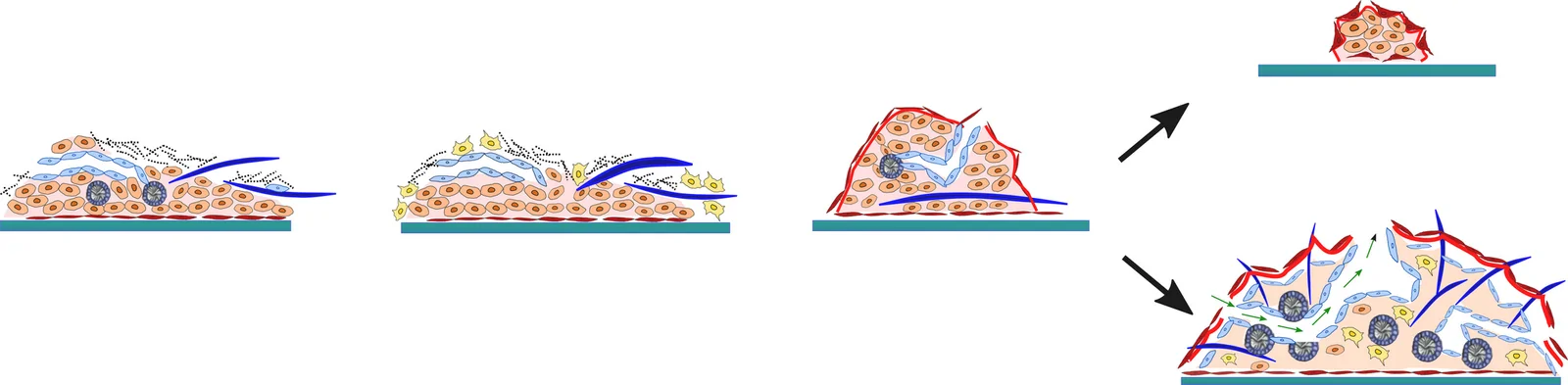
Concluding graphic of the stages of ectosome formation during whole-body regeneration and the stress-resistant form. Ectosome regeneration initiates when the choanosome is exposed. Collagenous extracellular matrix (*black dashed lines*) is deposited on the surface, and pinacocytes (*blue cells*) initiate migration and a mesenchymal-to-epithelial transition. When cultivated under unfavorable conditions, the microexplant internal region (*light pink*) transits to a compacted state covered by the formed exopinacoderm (brown epithelial cells) over a layer of extracellular matrix (*red line*). After complete WBR, transversal spicules projecting outward are organized to sustain the newly formed subdermal space. Choanocyte chambers and endopinacoderm-forming internal water channels are indicated in *blue*.

As summarized in Fig. 8, extracellular deposition is suggested as the first condition for mesenchymal-epithelial transition and pinacoderm epithelization. After, the spicules were transversally organized, and it allowed subdermal space formation when the choanosome became functional. Different adhesion to a common extracellular matrix orientates epithelial and mesenchymal cell morphology in demosponge.

## Discussion

A series of programmed events for ectosome regeneration was previously discussed (Borisenko *et al*. 2015; Ereskovsky *et al*. 2020, 2021; Lavrov *et al*. 2025). According to these authors, deposition of a thick collagen layer was reported at the initial stage, which was proposed for clotting and isolating the exposed area of the forming ectosome. This initial deposition of collagen, followed by the emergence of a new epithelial cell layer, was also observed in microexplants deprived of ectosome. The surface in the first stage of microexplant regeneration is then covered by exopinacocytes, as described previously (Ereskovsky *et al*. 2020). Our observations were not sufficient to distinguish the origin of the exopinacocytes; however, new exopinacocytes per se can emerge only through transdifferentiation, either from the endopinacoderm or from sponge stem cells of the choanosomal cell mass. The exopinacoderm can rely on two mechanisms to further complete the formation of a covering layer: (1) flattening and extension of already differentiated exopinacocytes and (2) further recruitment of choanosomal cells into the transdifferentiation (Ereskovsky *et al*. 2020). The gaps observed on day 3 microexplants may indicate exopinacoderm rearrangement with increased collective cell migration and exopinacoderm disturbance. The pinacocyte epithelial-mesenchymal transition is induced by collective mesenchymal cell migration (Coutinho *et al*. 2017 and Costa *et al*. 2020) and probably causes these gaps in the exopinacoderm. In addition, these gaps are devoid of fibrous extracellular matrix, which should induce and sustain exopinacocytes. The microexplant’s compacted morphology and the evidence of oriented collective migration may also indicate incomplete regeneration and persistent choanosomal remodulation. Patches of pinacoderm formation, comprising the extracellular matrix at its base, indicate that there is no initial place for pinacoderm formation in microexplants, as expected. Moreover, epithelization is triggered by the exposed choanosome, and exopinacocytes emerge from the choanosomal region without an increase in cell mass, as reported in Lavrov *et al*. 2025.

Pinacocytes in regenerating microexplant takes support of the collagen mesh to form the superficial exopinacoderm within the first 2 d of regeneration, consistent with the previously described regenerative stages 1 for ECM deposition and stage 2 for the pinacoderm formation by Ereskovsky *et al*. (2020) and Ereskovsky *et al*. (2021) and 1–3 d in primmorphs (Lavrov *et al*. 2025). Contrasting with the sponge class Homoscleromorpha and all bilaterians, demosponges are deprived of collagen IV and laminin, which form the lamina basal to instruct the epidermis formation and maintenance. The collagen mesh basing pinacoderm in demosponges would be a more ancestral feature of the Homoscleromorpha basement membrane (Gazave *et al*. 2012). The collagen mesh basing pinacoderm is not structurally distinct from the mesohyl collagen matrix; however, the asymmetrical adhesion to the ectosomal extracellular matrix results in the flattened morphology of pinacocytes, and the mesohyl collagen organization results in ameoboid/spherical morphology. In our analysis, amoeboid mesenchymal cells have more symmetrical adhesion and spherolous cells less adhesion. As an ancestral feature, the demosponge matrix was essential for establishing basic epithelial and mesenchymal cell states, and it later evolved into more specialized matrices for epithelium and mesenchyma, such as collagen IV in lamina basal. The hypothesis is the existence of a common primordial collagen matrix for epithelium and mesenchyme in last common ancestral of metazoans.

The spicules of the forming ectosome become trapped within the collagen extracellular matrix during the first 2 d. Once the microexplant is fully covered by the pinacoderm layer and favorable conditions are met, the spicules of the ectosome align transversely to the surface, with their tips protruding regularly toward the newly formed pinacoderm, which features ostia at this stage. The rising spicules are probably caused by the pulling of the collagen matrix, which supports the emerging subdermal space, akin to “poles supporting a circus tent,” as previously described by Nakayama *et al*. (2015) for the sponge *Ephydatia fluviatilis.* At this stage, after at least 4 d of regeneration, the microexplant is considered at an intermediate–adult stage, since it is still deprived of an osculum, which takes at least 7 d to appear (personal observation).

The emergence of subdermal space is the last stage of ectosomal regeneration, as now evidenced by the microexplant model. An equivalent process may be occurring for the digitate outgrowth reported for *Haliclona sp*. (Wanick *et al*. 2017). The expanding tip of the growing *Haliclona sp*. is deprived of subdermal space because longitudinal collagen fibers are anchored to the internal face of the pinacoderm. No water channels or choanocyte chambers were reported in the expanding tip of the digit, and the authors suggested this is an unmatured region. In contrast, subdermal space, feeding structures and water channels were observed in older digit proximal regions. A similar process was noticed by Nakayama *et al*. (2015) investigating ectosomal spicule rising in *Ephydatia fluviatilis.* Now we are reporting the same process of rising ectosomal spicules in the microexplant extending over the substrate (Fig. 6) and for the regeneration of the ectosome in the microexplant (Fig. 5). The common cell processes of subdermal space formation with rising ectosomal spicules reported for ectosomal final maturation in regeneration and growth suggest a pleiotropic modular process committed to tissue maturation.

Mesenchymal crawling cells and clusters of spheroid cells were observed on the outer part of the pinacoderm in both wild-type samples and regenerating microexplants. However, further investigations are necessary to confirm whether these are *Hymeniacidon heliophila* cells or protist epibionts. The presence of spherulous-mesenchymal cells on the surface of sponges has been previously documented by Vacelet (1967) and Ereskovsky *et al*. (2020), who demonstrated their extensive expulsion during surface healing. This expulsion of spherulous-mesenchymal cells to the exterior is a behavior also observed in undamaged sponges, likely to serve as a mechanism for releasing chemical defenses (Uriz *et al*. 1996; Ternon *et al*. 2016). *Hymeniacidon heliophila* is an encrusting sponge found in the intertidal zone of eutrophic tropical waters, exhibiting tolerance to high densities of epibionts and debris on its pinacoderm. The presence of external cells could be epibionts or external sponge cells essential for both nutrition and defense, and further investigations should address this. 

The formation of new individuals from microexplants aligns with observations commonly reported for surface ablation, primmorph formation, and explant cultivation in demosponges with leuconoid aquifers systems (Borisenko *et al*. 2015; Coutinho *et al*. 2017; Ereskovsky *et al*. 2020; Ereskovsky *et al*. 2021 and Riesgo *et al*. 2022; Lavrov *et al*. 2025). For instance, processes of choanosome disassembling and reassembling first reported in microexplant by Coutinho *et al*. 2017 align with collective cell migration and remodeling shown in Fig. 1. The emergence of the pinacoderm, oriented spicules with subdermal space (epithelization), and the eventual emergence of a functional osculum corroborates the current literature on programmed events of the ectosome formation. The ectosome repair processes may involve dedifferentiation and transdifferentiation of cells (Lavrov *et al*. 2025) and activation of a stereotype genetic program, as discussed in evolution of regeneration in animals (Elchaninov *et al*. 2021).

Whole-body regeneration (WBR) is an attribute of sponges, and several sponge-specific features contribute to it. For instance, the plasticity in remodeling their structures in response to environmental conditions (Simpson 1984) and the significant body changes during their life cycle, such as metamorphosis, are unifying traits in organisms capable of somatic embryogenesis (Alibardi 2023). Moreover, asexual reproduction, such as fragmentation, budding and gemmules, is associated with a high ability to WBR (Elchaninov *et al*. 2021). The primitive immune system in sponges may also contribute to it (Elchaninov *et al*. 2021). In contrast to sponges, which are not susceptible to cancer (Aktipis *et al*. 2015), the immune system in other animals eliminates non-differentiated cells initiating cancer along with foreign cells. The constant immune pressure on the populations of cells with high differentiation potential negatively affects the regenerative capacity (Godwin *et al*. 2017). Another feature contributing to sponge WBR is constant predation and the aggressive environmental conditions. This scenario was probably the selective pressure for the maintenance of the regenerative capacity in sponges. Indeed, a high frequency of damage is typical for some groups of highly regenerative organisms in natural environments (Wulff 2006).

As initially proposed by T. H. Morgan, the blastema is a mass of proliferating undifferentiated cells that forms beneath a wounded area during epimorphic regeneration, as seen in limb regeneration (reviewed in Elchaninov *et al*. 2021). In contrast, morphallaxis involves the remodeling of preexisting structures without cell proliferation, a process exemplified by regeneration in *Hydra* (Sánchez 2000). Epimorphosis and morphallaxis are mainly distinguished by the scale of damage-induced cell proliferation and its contribution to morphogenesis. The inclusion of trans-differentiation in regeneration scenarios, along with the observation of organisms mostly exhibiting features of both epimorphosis and morphallaxis, has blurred the boundaries between these two processes. Many authors now tend to avoid these terminologies, as discussed in Riesgo *et al*. (2022), Agata *et al*. (2007), and Bely and Nyberg (2010). According to Agata *et al*. (2007) and Candia Carnevali (2006), significant similarities between epimorphosis and morphallaxis suggest that these processes are evolutionarily related. The broad representation of morphallaxis in both bilaterians and non-bilaterians, whereas epimorphosis is specific to bilaterians, indicates the phylogenetic primacy of morphallaxis (Bely and Nyberg 2010). Considering their similarity, it can be assumed that epimorphosis evolved on the basis of morphallaxis (Agata *et al*. 2007; Ben Khadra *et al*. 2018; Ferrario *et al*. 2018).

Both regeneration mechanisms have been described in sponges, blastema in demosponges and tissue remodeling (morphallaxis) in calcarean/Homoscleromorpha, (Ereskovsky *et al*. 2020, 2021). Recently Lavrov *et al*. (2025) reconsidered WBR in demosponges *Halisarca dujardinii* based on new interpretation of the dynamic process of cell proliferation and cell death. The no significant cell proliferation in demosponge undifferentiated cell mass, previously interpreted as blastema by Ereskovsky *et al*. (2020), was critical for Lavrov *et al*. (2025) to consider the dedifferentiate cell mass as the result of tissue remodeling. This agrees with previous research on sponge regeneration, as reviewed in Alexander *et al*. (2015). Morphallactic in *Halisarca dujardinii* restorative process would rely primarily on cell migration, dedifferentiation, and trans-differentiation. Cell proliferation was not quantified in microexplant WBR, and the extension of morphallaxis or epimorphosis cannot be conclusive without cell proliferation assay. However, no increase in cell mass was observed during WBR microexplant, but a complete remodulation of the tissue.

Contrasting with remodulation in morphallaxis, the blastema contains key components of molecular pathways regulating the establishment of anterior–posterior (Wnt-signaling), dorsal–ventral (BMP-pathway), and medial–lateral polarities (Sánchez Alvarado 2000; Elliott and Sánchez Alvarado 2013; Karami *et al*. 2015). Basal–apical polarity is preserved when microexplants initiate regeneration and is maintained throughout the entire formation of the adult individual. As expected, the apical–basal polarity is insufficient to retain the original information of the water flow axis, since the axis is formed de novo during the final stage of regeneration. Considering the similar Wnt-signaling for anterior–posterior axis and sponge water flow axis establishment, its disorganization contrasts with axis instructions in the blastema, thus supporting morphallaxis.

The emergence of the water flow axis occurs only after the formation of a dense choanosome, despite the fully preserved apical–basal axis. Ereskovsky *et al*. (2021) and Riesgo *et al*. (2022) pointed to adhesion to the substrate and the establishment of apical–basal polarity as critical for the formation of the ostia-osculum axis after a dense primmorph-like stage. The current investigation with a microexplant confirms the conclusion that a dense choanosome with an apical–basal oriented axis acts as a prerequisite stage for the subsequent development of the ostia-osculum body axis.

The investigation of the ectosomal regeneration in microexplants revealed an evolutionarily primordial process in metazoan regeneration. The first stage, with deposition of a collagenous extracellular matrix at the microexplant’s external surface guiding epithelial cells, is probably homologous to the fibrosis process during metazoan wound healing. As a primordial feature of the epithelium and mesenchyma, the composition of the demosponge collagenous extracellular matrix is not distinct from that of the mesenchymal collagenous matrix. Moreover, the flattened or three-dimensional extracellular matrix mesh organization controls epithelial and mesenchymal cell morphology. The ectosomal regeneration has stages equivalent to those of the growth process, revealing a modular commitment to sequential programmed cell processes for growth and regeneration.

## Data Availability

The data that support the findings of this study are available from the corresponding author upon reasonable request.
